# Using Concept Maps to compare obesity knowledge between policy makers and primary care researchers in Canada

**DOI:** 10.1186/s13104-018-4042-x

**Published:** 2019-01-14

**Authors:** Elizabeth Sturgiss, Thea Luig, Denise L. Campbell-Scherer, Richard Lewanczuk, Lee A. Green

**Affiliations:** 1 Epidemiology for Policy and Practice Group, National Centre for Epidemiology and Population Health, Research School of Population Health, The Australian National University, 62 Mills Road, Acton, ACT 2601 Australia; 2 Department of Family Medicine, University of Alberta, Edmonton, AB T6G 1C9 Canada; 3 Department of Medicine, University of Alberta, Edmonton, AB T6G 2B7 Canada; 4 Department of General Practice, Monash University, Building 1, 270 Ferntree Gully Road, Notting Hill, Melbourne, 3168 Australia

**Keywords:** Obesity, Primary care, Research, Knowledge translation, Health policy, Canada

## Abstract

**Objective:**

Knowledge transfer is the process of information sharing between researchers, knowledge users and policy makers. Globally, public policies about obesity do not reflect the complexity of what is known about the cause and effects of obesity. We used Concept Maps, a qualitative method that represents mental models, to compare the understanding of obesity between policy makers in a Canadian province and local primary care researchers. Eight participants were interviewed during which a Concept Map was developed using “C-map Tools” software. Maps were then colour-coded to identify themes and concepts in the maps. Finally, the team synthesised the findings from each of the maps and presented them back to each of the participants.

**Results:**

All participants had mental models with rich details on the complexity of obesity for individuals, community, and at the policy level. Clinician-researchers had more focus on medical management than policy makers although most participants lacked concepts on the role of primary care in obesity management. A shared understanding of obesity could assist researchers and policy makers in developing a relevant and effective strategy. Concept Mapping provides a novel and creative way to visually compare different understandings of health-related topics.

**Electronic supplementary material:**

The online version of this article (10.1186/s13104-018-4042-x) contains supplementary material, which is available to authorized users.

## Introduction

Obesity is a global problem that affects individuals’ quality of life, challenges the way communities live, and is an urgent priority for health policy makers [1]. Obesity is a complex disease [2] that is influenced by a broad range of biomedical, social, and environmental factors [3]. Collaboration between the health, policy, and research sectors is required for any progress across populations [4, 5]. Unfortunately, the burden of disease from obesity across Canada continues to increase with ongoing challenges for patients in accessing care [6].

Knowledge transfer (KT) is the process of information sharing between policy makers, researchers, knowledge users, and industry leaders [7, 8]. It is an essential part of the research process as effective KT helps to ensure return on research investment [9]. All areas of research need improvements in KT [10], and our research was specifically interested in improving KT for the management of obesity in the primary care setting.

The understanding that researchers and policy makers have of a subject area influences the KT process [11, 12]. For example, what does each group think about the root causes of disease, how these causes can be interrupted or managed, and what outcomes are expected from interventions? Without a shared vision of these basic concepts it is unlikely that researchers and policy makers would arrive at a common view on the best way to progress a health issue. This project uses a creative and interactive method to compare and contrast the mental models of obesity between policy makers and clinician-researchers.

Concept Mapping is a form of Cognitive Task Analysis that has been used successfully in a variety of settings (i.e. education, healthcare, military, space exploration) to represent expert knowledge in a way that others can understand [13]. Concept Maps are useful for comparing the “mental models” that different people have about the same topic. A mental model is the ever evolving, working understanding a person has of a subject area [14]. It includes the basic knowledge and facts, the processes and links between each of the working parts, and reflects the person’s beliefs and values about a topic. A mental model is also the reference base for a person trying to learn new skills or to adopt a new approach [14].

For example, primary care researchers will have a certain mental model of obesity that will shape their work in the area. Similarly a policy maker will use their mental model of obesity to make decisions about health policy. If two groups have different mental models about the same topic, KT becomes difficult as neither group can conceptualize the others’ viewpoint [11]. This can lead to delays and deferments in the process of negotiating new health innovations.

This project used Concept Mapping to show differences and similarities in how primary care researchers and health policy makers think about obesity.

## Main text

This project was approved by the University of Alberta Human Research Ethics committee Pro00074651.

We purposefully sampled individuals from two specific groups:

1. Health policy makers who have input into the policy agenda and decision making in their organization.
2. Researchers who are developing interventions for obesity management in primary care.

Participants were invited through the local health policy division and the local obesity research networks via email from the research team. Formal written consent was taken at the start of the interview.

Concept mapping is a formal technique requiring two interviewers and the use of “C-map Tools” (https://cmap.ihmc.us/cmaptools). Concept Maps visually represent knowledge and how a person links knowledge concepts [15]. Each concept is placed in a box and between each box is a linking statement that defines the relationship between the two concepts. Boxes at the top of the map represent higher order concepts and related concepts are linked down through the diagram. Comparing Concept Maps from different individuals allows researchers to gain insight into various mental models on the same topic [15].

Two members of the research team (ES, TL) conducted all interviews either face-to-face or online, depending on the preference of the interviewee. One researcher (TL) asked questions and the second facilitator (ES) took notes and constructed the Concept Map. Interviews lasted about 1 h and were audiotaped with the participant’s permission.

**Fig. 1:**
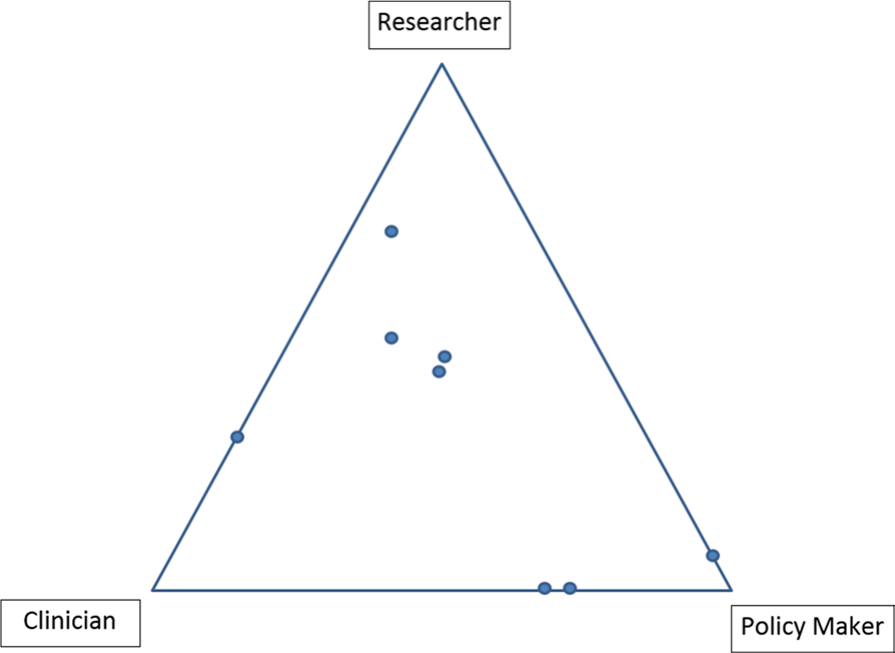
Self-identification by participants of their professional role during their Concept Mapping interview

Participants were asked to mark on a triangle (Fig. 1) their professional role. As dictated by the concept mapping technique, interviews began with a specific focus question (“Tell me what you know about obesity”) followed by prompts based on a semi-structured guide. At the same time, the second facilitator entered concepts as mentioned by the interviewee and linkages between concepts into C-map and constructed a preliminary concept map. After approximately 30 min, the first interviewer wrapped up the interview and then second interviewer asked additional questions for clarification. Then the preliminary map was shown to the interviewee and they were asked to sort and re-order concepts and linkages to accurately reflect their mental model of the topic. After the session participants were forwarded their Concept Map to allow them to make any final comments or changes.

ES and TL then compared concepts within and across maps to identify commonalities and differences in the mental models about obesity. ES and TL used colour codes to represent the different themes that were discussed in the interviews. Colour coded maps were presented to the rest of the research team to compare the concepts and synthesise the findings. These findings were relayed to all participants via email immediately after this meeting.

### Results

**Fig. 2:**
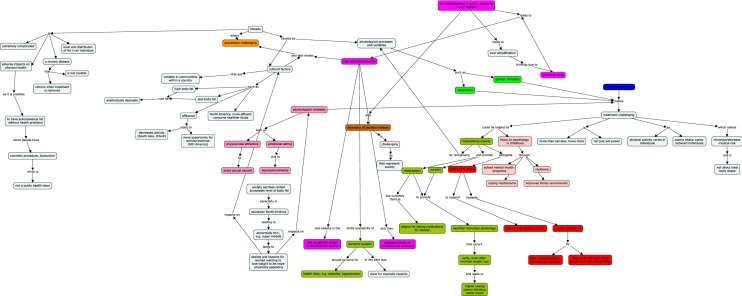
Example of a Concept Map developed during the interview with a participant. Bright pink—weight bias and stigma; red—policy; olive green—medical management; bright green—genetics; light pink—childhood events; brown—education; blue—social determinants of health

There were eight participants—three identified predominantly as policy makers, two were clinician-researchers, and three had roles that were almost evenly distributed between the three categories. An example of a Concept Map is shown in Fig. 2 and all Concept Maps are shown in Additional file 1: Map 1, Additional file 2: Map 2, Additional file 3: Map 3, Additional file 4: Map 4, Additional file 5: Map 5, Additional file 6: Map 6, Additional file 7: Map 7 and Additional file 8: Map 8.

#### Policy makers

Mental models of participants with a policy role revolved around managing population level drivers to improve obesity and did not include medical management. They included policy to improve social determinants of health, working towards prevention with programs targeting children in school and community, education of public and health professionals, and addressing stigma. Policy makers rarely mentioned the role of medications and bariatric surgery in the management of the obesity.

#### Clinician-researchers

Participants with a clinical role placed more emphasis on factors at the individual level. When speaking about management options, clinicians had mental models that aligned with person-centred care—meaning management that builds on the clinician’s understanding of biological drivers of obesity and how a patient’s lifecycle and life events impact their health. Management options included addressing functional impairment, medication, bariatric surgery, and improving overall well-being and resilience through the use of interdisciplinary healthcare providers.

#### Comparison

All the Concept Maps demonstrated a shared understanding of obesity as a complex and chronic condition. It was clear from the colour coding that all had a broad understanding of the issues involved in obesity from the individual level to the broader social environment. The identified root causes were wide ranging—genetic factors, life events and mental health, interactions between individual and environmental factors that shape nutrition and access to space for physical activity, as well as the wider community’s discourse of beauty and body acceptance (Fig. 2). Every participant had multiple different themes in their map—no one had only one area, or even a majority of one area. All participants had a mental model of obesity that is in line with current understanding of the biology, treatment, and prevention of obesity.

Many participants emphasized the need for a shift in policy, healthcare funding, research funding, and medical training to better respond to the complexity of obesity. However, the maps did not include policy that would enhance medical management of obesity and the organization of care. Overall, it was rare for participants to highlight the role of primary care in managing or preventing obesity.

### Discussion

The Concept Maps were a useful way to compare the mental models of obesity between policy makers and researchers. The colour coding of the themes in each map made the comparison easier as the similarities and differences in the large maps could be visualized by the research team.

The rich, diverse, and detailed mental models of all participants reflected the current understanding of obesity pathophysiology and management. Not surprisingly, the policy makers focused more on population themes and those with a clinical background put more emphasis on individual management. Overall, the maps illustrated the participants’ understanding of the need for better linkages and cooperation between healthcare, research, policy, and community resources.

This is the first time, that we are aware of, that Concept Maps have been use to compare mental models between researchers and policy makers. This technique could be used in both the research setting and policy arena to determine if all team members are on the same page. The technique can also be used in team settings—where the team develops a Concept Map together allowing differences in understanding to be exposed. A lack of shared understanding of concepts could be a significant barrier to knowledge transfer between researchers and policy makers [16]. Personal interaction between policy makers and researchers assists knowledge transfer [17] and team concept mapping could be a method for increasing interaction and active engagement.

There is compelling evidence that relationships and personal contact between policy makers and researchers facilitates the use of research evidence in policy decision making [16]. This is likely to be due to reducing mistrust between the two groups. Further, early engagement with policy makers can ensure that research questions are framed in a way that is useful for policy makers [18] plus the experience of policy makers can be incorporated into the resulting research [16]. Creative methods to assist shared understanding and enhance personal interactions could be useful for improving research based policy-making.

We found the triangle for participants to self-identify their professional role very useful (Fig. 1). It made it clear how the person saw their role and the likely influence they had on policy-making and research. It was useful to group the maps into those with similar roles and by doing this we saw the pattern between populations versus individual focus. We would recommend using this technique in qualitative research particularly when aiming to recruit people with differing roles.

The Concept Mapping process was very valuable compared to regular interviews with thematic coding done after the interview. As the Concept Map is made during the interview, the themes are checked with the interviewee during the process. This also meant less research labour was needed after the interview in contrast to traditional thematic analysis approaches to interview data. Analysis occurred through colour coding the maps and discussing emergent themes and patterns during team meetings. This could be a potential benefit of this method for answering similar research questions.

## Limitations

We noticed poor alignment between the richness of all participants’ mental models and prevailing policies that do not sufficiently address the complexity of obesity to support prevention and management [4, 19]. It is likely that our self-selected group had a prominent interest in obesity and a deeper understanding of its complexity. Decision making in the policy arena usually occurs by committee consensus—although there were policy makers participating in this project, it is possible that their voices are not heard, or are over-ruled by alternative viewpoints on the committees they are involved in. This project did not investigate the process of decision making about obesity policy that the participants are involved in.

## Additional files

**Additional file 1: Map 1.** Concept Map from a clinician/researcher. Bright pink—weight bias and stigma; red—policy; olive green—medical management; bright green—genetics; light pink—childhood events; brown—education; blue—social determinants of health.

**Additional file 2: Map 2.** Concept Map from a clinician/researcher/policy maker. Bright pink—weight bias and stigma; red—policy; olive green—medical management; bright green—genetics; light pink—childhood events; brown—education; blue—social determinants of health.

**Additional file 3: Map 3.** Concept Map from a clinician/researcher/policy maker. Bright pink—weight bias and stigma; red—policy; olive green—medical management; bright green—genetics; light pink—childhood events; brown—education; blue—social determinants of health.

**Additional file 4: Map 4.** Concept Map from a policy maker/clinician. Bright pink—weight bias and stigma; red—policy; olive green—medical management; bright green—genetics; light pink—childhood events; brown—education; blue—social determinants of health.

**Additional file 5: Map 5.** Concept Map from a policy maker/clinician. Bright pink—weight bias and stigma; red—policy; olive green—medical management; bright green—genetics; light pink—childhood events; brown—education; blue—social determinants of health.

**Additional file 6: Map 6.** Concept Map from a policy maker/researcher. Bright pink—weight bias and stigma; red—policy; olive green—medical management; bright green—genetics; light pink—childhood events; brown—education; blue—social determinants of health.

**Additional file 7: Map 7.** Concept Map from a clinician/researcher/policy maker. Bright pink—weight bias and stigma; red—policy; olive green—medical management; bright green—genetics; light pink—childhood events; brown—education; blue—social determinants of health.

**Additional file 8: Map 8.** Concept Map from a policy maker/clinician. Bright pink—weight bias and stigma; red—policy; olive green—medical management; bright green—genetics; light pink—childhood events; brown—education; blue—social determinants of health.

## Abbreviation

KT: knowledge transfer
