# Supplementary figures and images for: Using Concept Maps to compare obesity knowledge between policy makers and primary care researchers in Canada
Source: BMC Res Notes. 2019 Jan 14;12:23. doi: 10.1186/s13104-018-4042-x (PMC6332696; doi:10.1186/s13104-018-4042-x)

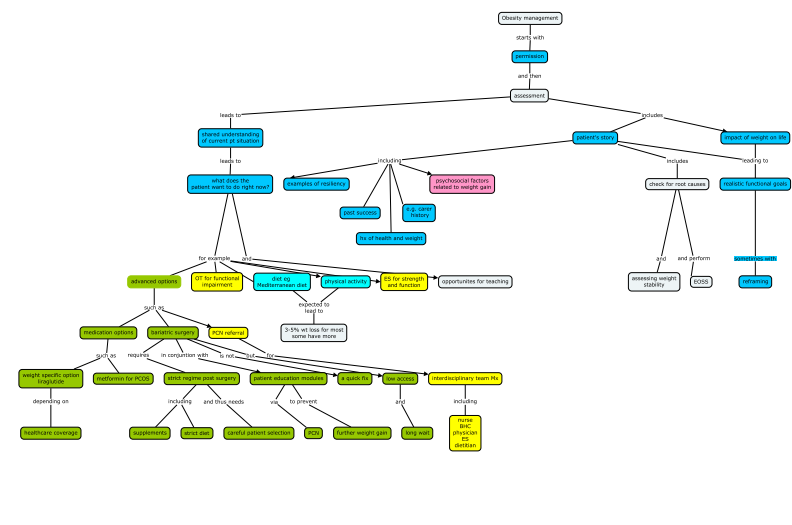

Supplement: Supplementary file 1 — Additional file 1: Map 1. Concept Map from a clinician/researcher. Bright pink—weight bias and stigma; red—policy; olive green—medical management; bright green—genetics; light pink—childhood events; brown—education; blue—social determinants of health. [file 13104_2018_4042_MOESM1_ESM.pdf]

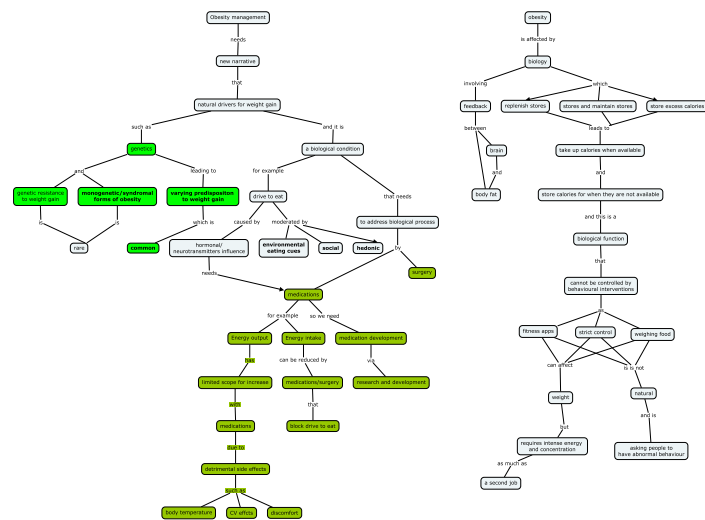

Supplement: Supplementary file 2 — Additional file 2: Map 2. Concept Map from a clinician/researcher/policy maker. Bright pink—weight bias and stigma; red—policy; olive green—medical management; bright green—genetics; light pink—childhood events; brown—education; blue—social determinants of health. [file 13104_2018_4042_MOESM2_ESM.pdf]

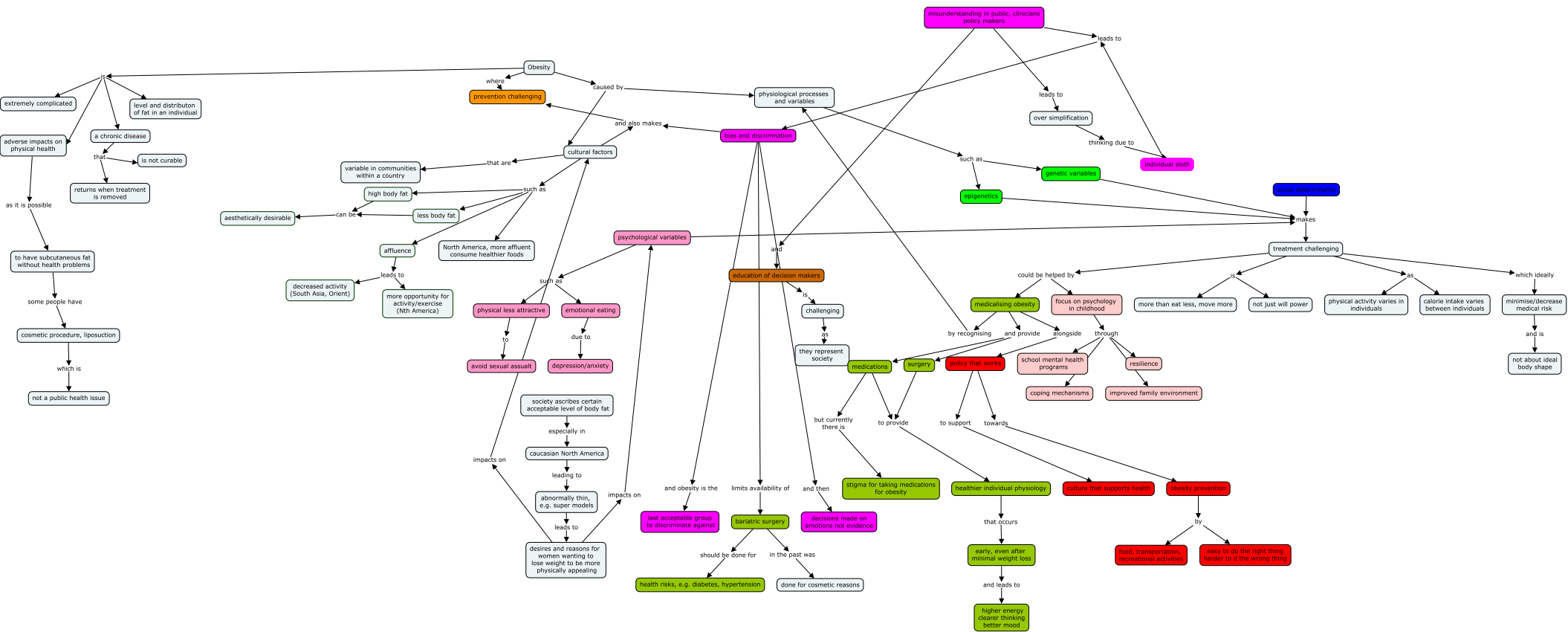

Supplement: Supplementary file 3 — Additional file 3: Map 3. Concept Map from a clinician/researcher/policy maker. Bright pink—weight bias and stigma; red—policy; olive green—medical management; bright green—genetics; light pink—childhood events; brown—education; blue—social determinants of health. [file 13104_2018_4042_MOESM3_ESM.pdf]

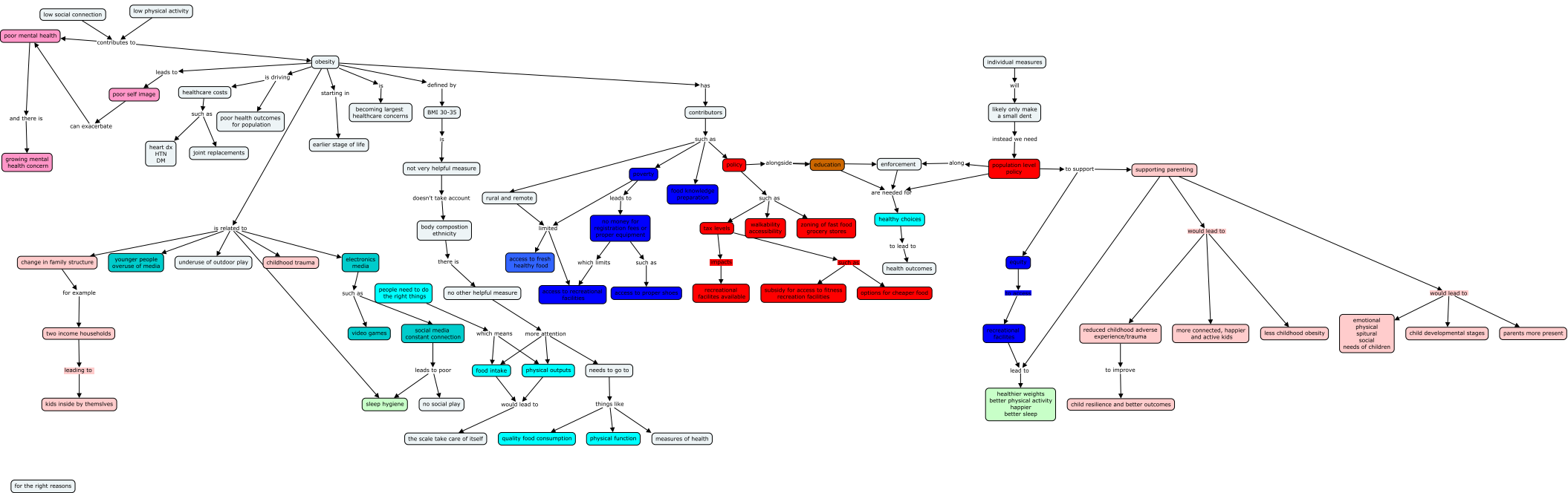

Supplement: Supplementary file 4 — Additional file 4: Map 4. Concept Map from a policy maker/clinician. Bright pink—weight bias and stigma; red—policy; olive green—medical management; bright green—genetics; light pink—childhood events; brown—education; blue—social determinants of health. [file 13104_2018_4042_MOESM4_ESM.pdf]

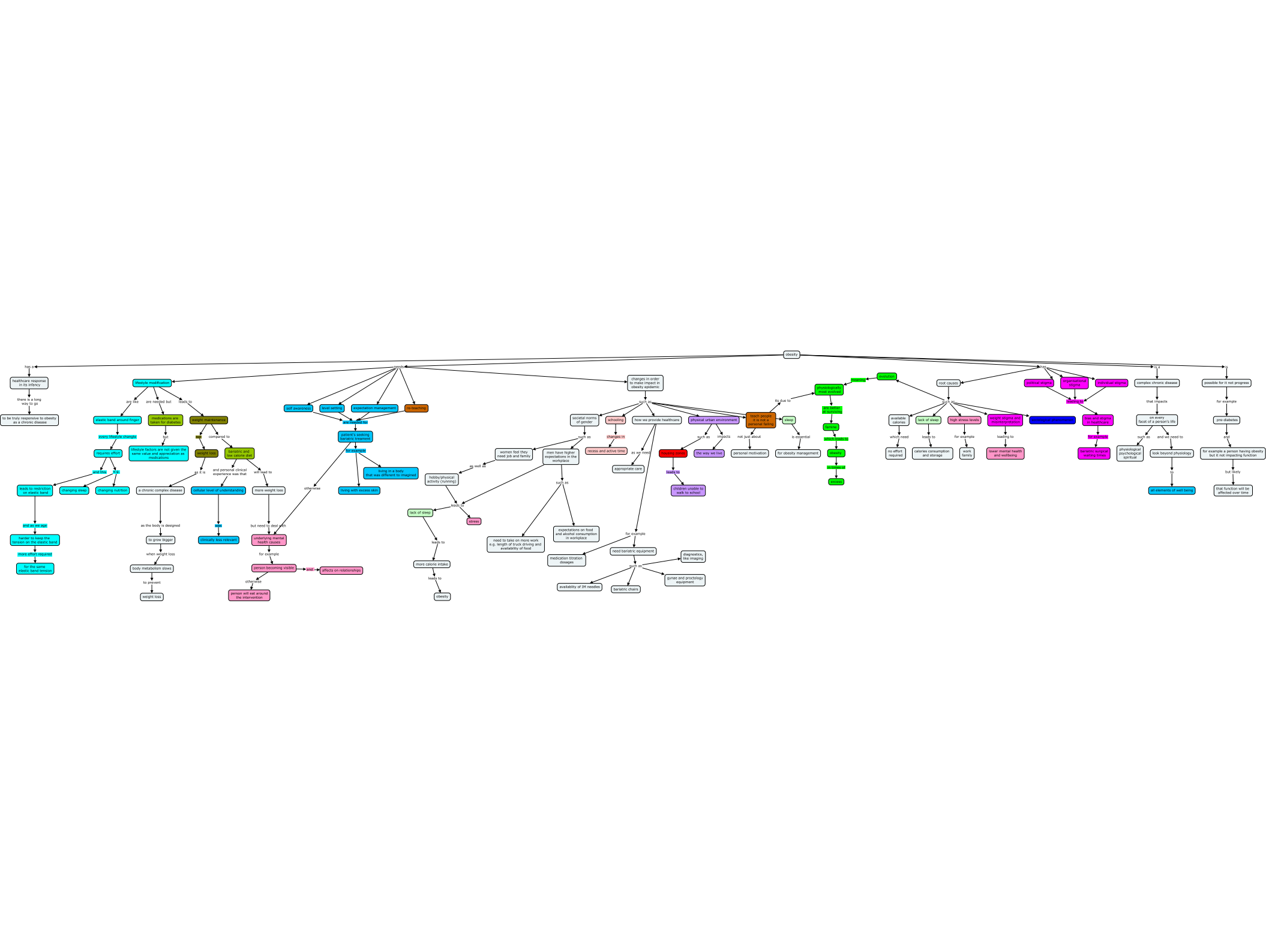

Supplement: Supplementary file 5 — Additional file 5: Map 5. Concept Map from a policy maker/clinician. Bright pink—weight bias and stigma; red—policy; olive green—medical management; bright green—genetics; light pink—childhood events; brown—education; blue—social determinants of health. [file 13104_2018_4042_MOESM5_ESM.pdf]

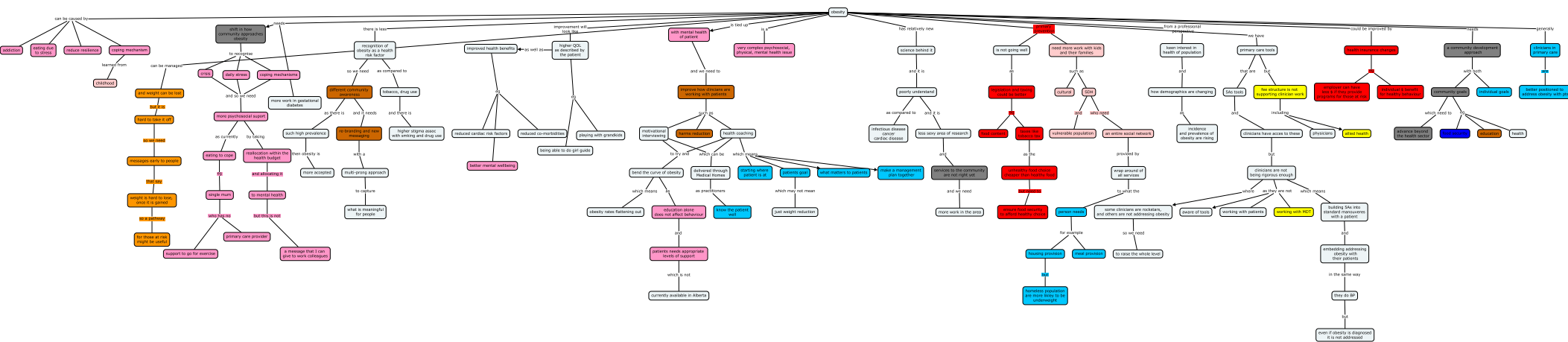

Supplement: Supplementary file 6 — Additional file 6: Map 6. Concept Map from a policy maker/researcher. Bright pink—weight bias and stigma; red—policy; olive green—medical management; bright green—genetics; light pink—childhood events; brown—education; blue—social determinants of health. [file 13104_2018_4042_MOESM6_ESM.pdf]

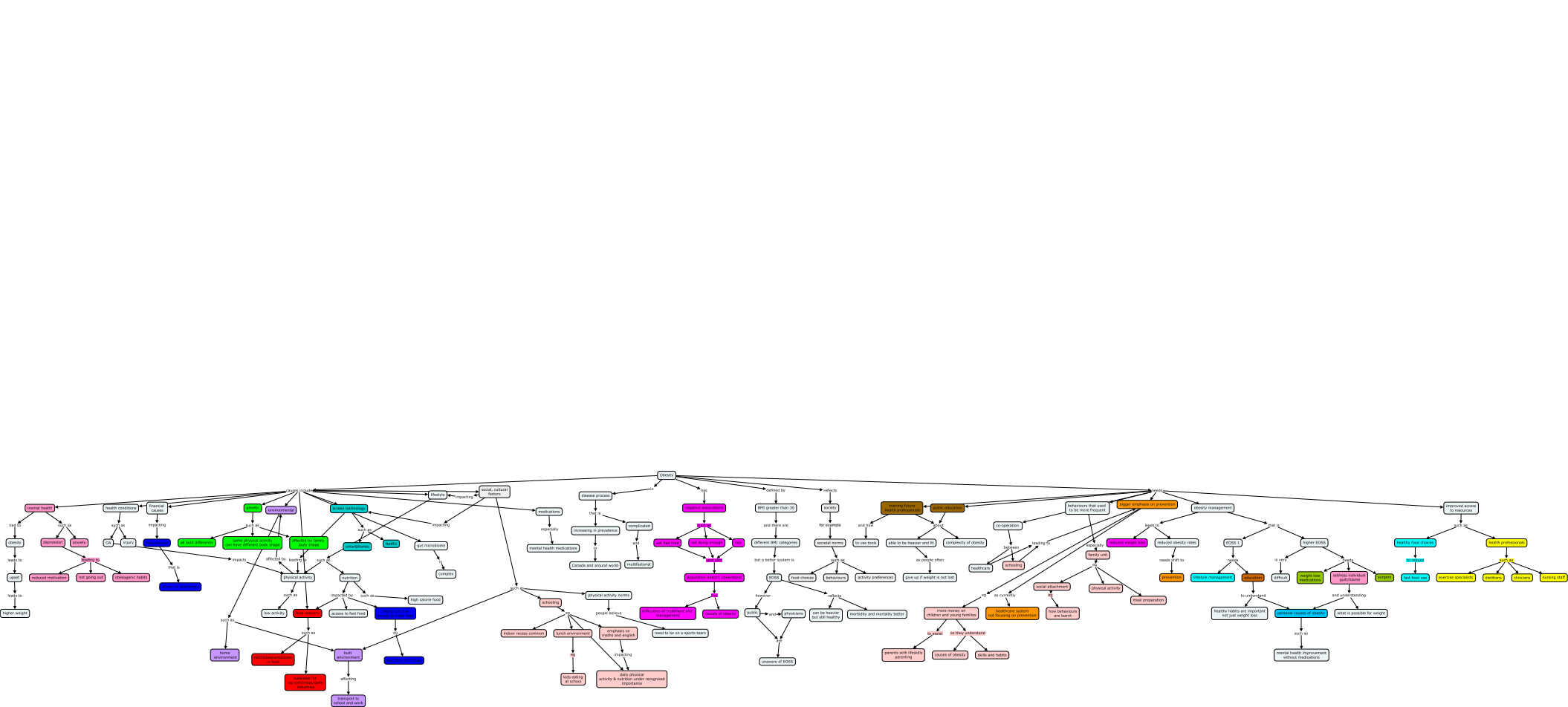

Supplement: Supplementary file 7 — Additional file 7: Map 7. Concept Map from a clinician/researcher/policy maker. Bright pink—weight bias and stigma; red—policy; olive green—medical management; bright green—genetics; light pink—childhood events; brown—education; blue—social determinants of health. [file 13104_2018_4042_MOESM7_ESM.pdf]

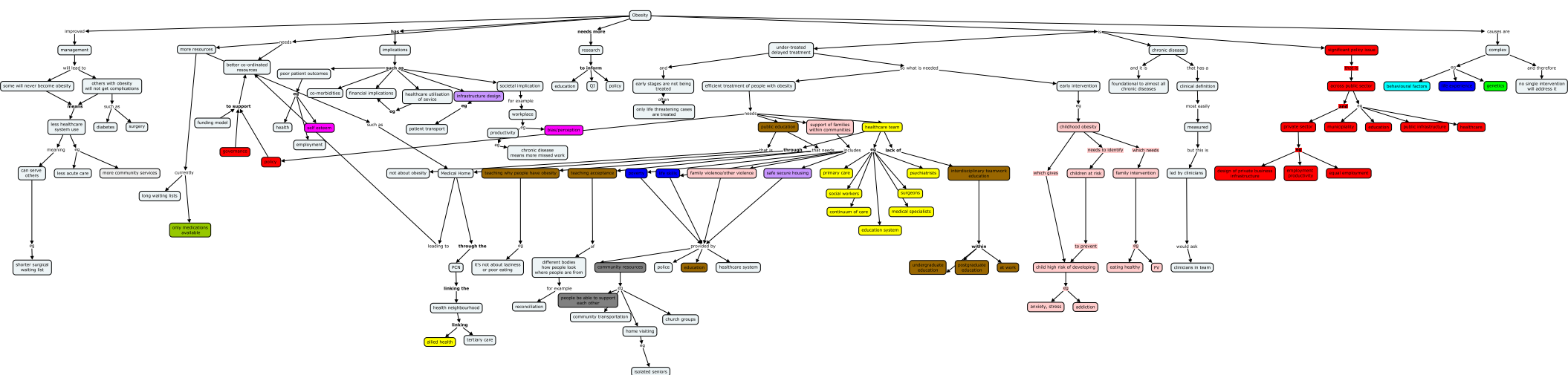

Supplement: Supplementary file 8 — Additional file 8: Map 8. Concept Map from a policy maker/clinician. Bright pink—weight bias and stigma; red—policy; olive green—medical management; bright green—genetics; light pink—childhood events; brown—education; blue—social determinants of health. [file 13104_2018_4042_MOESM8_ESM.pdf]
